# A Comparative Analysis of the Universal Elements of Music and the Fetal Environment

**DOI:** 10.3389/fpsyg.2016.01158

**Published:** 2016-08-09

**Authors:** David Teie

**Affiliations:** School of Music, University of Maryland, College Park, College ParkMD, USA

**Keywords:** origin of music, womb, pulse, music, rhythm

## Abstract

Although the idea that pulse in music may be related to human pulse is ancient and has recently been promoted by researchers (Parncutt, 2006; Snowdon and Teie, 2010), there has been no ordered delineation of the characteristics of music that are based on the sounds of the womb. I describe features of music that are based on sounds that are present in the womb: tempo of pulse (pulse is understood as the regular, underlying beat that defines the meter), amplitude contour of pulse, meter, musical notes, melodic frequency range, continuity, syllabic contour, melodic rhythm, melodic accents, phrase length, and phrase contour. There are a number of features of prenatal development that allow for the formation of long-term memories of the sounds of the womb in the areas of the brain that are responsible for emotions. Taken together, these features and the similarities between the sounds of the womb and the elemental building blocks of music allow for a postulation that the fetal acoustic environment may provide the bases for the fundamental musical elements that are found in the music of all cultures. This hypothesis is supported by a one-to-one matching of the universal features of music with the sounds of the womb: (1) all of the regularly heard sounds that are present in the fetal environment are represented in the music of every culture, and (2) all of the features of music that are present in the music of all cultures can be traced to the fetal environment.

## The Question of the Origins of Music

It is reasonable to postulate that the characteristics of music that are common to the music of all cultures must have a common origin. Is there a correlation between the acoustic features of the common elements of music and the acoustic features of sounds that are present in the natural world? If so, is there a consistent and universal aspect of human development that would allow those sounds from the environment to be implanted as templates of recognition in the brains of peoples of diverse and widely separated cultures? Finally, if that common environment is prenatal, does the neurological development of the fetus allow for the absorption and retention of information from the womb? If the prenatal acoustic environment contains characteristic sounds that are the bases for some of the elements of music, then it should be possible to match, one-for-one, the sounds of the womb to those elements that can be found in the music of all cultures.

This hypothesis is not intended to be complete nor exclusionary. The scope is restricted to the elements of music that may be traced to the sonic environment during fetal development; it does not attempt to address the various expressions and uses of music or narrative expression such as those that are found in social connections, communicative support, kinetic synchrony, and motivation. A variety of well-supported explanations for our enjoyment of music such as expectation, maternal and social bonding, and development of narrative through shared activity may be understood as parallel to and not contrary to the bases outlined below. The proposition that the origin of the common elements of music may be found in the sonic environment of the womb is not inconsistent with a host of other valid premises and observations concerning the developments of the many culturally diverse languages of music.

## Outline of Hypothesis

This outlines a proposal that there is a fetal origin of the fundamental building blocks of music. The sounds heard by the fetus for four months before birth may permanently etch the foundations of music into the collection of brain structures that form what is commonly known as the limbic system. These structures are primarily responsible for our emotions and are almost fully formed at birth (Huang et al., 2006). The features of fetal development that make it possible for the fetus to hear sounds, remember (subconsciously) those sounds in adulthood, and respond to them emotionally have been demonstrated in studies that are cited below. When these features are added together and compared to the construct of music it is reasonable to conclude that the part of our brains that is responsible for emotions retains information from and recognizes the sonic environment of the womb.

The prevalent sounds of the fetal environment are pulse, respiration, footfalls, and the mother’s voice. McDermott (2008) identified several universal properties of music: pulse, hierarchal organization of scales (tonality), infant-directed song, dance, and meter. To McDermott’s list I propose adding: amplitude contour of pulse instrument, prevalence of discrete single-frequency units (musical notes), varied pitches and rhythms in the melodies (prosody), the 200–900 Hz frequency range of melodic instruments, and continuity. The following is an outline of the musical elements that are based on the sounds of the womb: pulse (pulse is understood as the regular, underlying beat that defines the meter), amplitude contour of pulse, meter, musical notes, elements related to prosody (syllabic contour, melodic rhythm, melodic accents, phrase length, and phrase contour), melodic frequency range, and continuity. All of these universal features of music can be traced to the womb and all of the sounds heard in the womb are represented in the music of all cultures. Table 1 outlines the sounds heard in the womb and the corollary features of music.

**Table 1:** Sounds of the womb and their corollaries in music.

Features of sounds heard in the womb:	Features of music found in all cultures:
Heartbeat, respiration, and footfalls	Pulse
Amplitude contour of pulse	Amplitude contour of pulse instrument
Combined heartbeat, respiration, and footfalls	Meter
Spoken syllables of the maternal voice	Musical notes
Prosody of maternal speech	Prosody of melodic phrasing
Frequency range of adult female voice	Frequency range of melodic instruments
Continuous	Continuous

## Limbic System Development Memory

In light of anatomical studies that have emphasized the interconnections between ventral limbic circuits and the motor control connections between striatum and motor cortex (Gunnar and Nelson, 1992), I propose that the acoustic information that pervades the development of structures responsible for our emotions as well as structures near the brainstem responsible for repetitive movement are the sources and origins of pulse, meter, and rhythm in music. The elements of music that are most universal are musical representations of sounds that are heard during the time when the limbic structures are formed in the developing brain. The following conditions allow for the formation of lasting fetal acoustic memories: The human fetus is able to hear at 24 weeks, providing 4 months of constant sound exposure (Birnholz and Benacerraf, 1983) prior to birth. The sound of the maternal heartbeat is 25 db above basal noise, dominating the fetal environment (Querleu et al., 1988). The maternal voice is heard in the uterus nearly four times more strongly than it is heard externally (Richards et al., 1992). In utero research and analysis has shown consistent evidence that the fetus responds to the sound of the mother’s heartbeat (Porcaro et al., 2006) and that infants also respond to the prosodic features of speech (see below).

The combination of three features of human fetal development make it possible for the sounds of the womb to provide a lasting template of recognition: (1) the dearth of competing sensory information in the fetal environment allows sound to be a primary source of varied and ever-present information entering the developing brain, (2) information that is well-organized when incoming while a brain structure is plastic will tend to remain organized in the brain, and (3) the limbic structures are well-developed at birth. 3D brain imaging technology used by scientists in Baltimore (Huang et al., 2006) showed that the structures of the limbic system are almost completely formed at birth. The limbic fibers, the cingulum and the fornix, are two of the most dominant tracts in the fetal brain and their entire trajectories are already developed at 19 gestational weeks. “Early formation of the limbic system is well known, and it is expected that limbic fibers are well formed at 19–20 gestational weeks.” – Huang et al. (2006, p. 36). The brain structures responsible for our emotions are able to retain information long before the upper cortical structures. A logical conclusion to the summed effects is that brain structures responsible for emotions and that are well developed at birth may remember and later respond to sounds that resemble those of the fetal environment.

## Pulse

A regular and repeated pulse is one of the universal traits of music is even though it is not found in human vocalizations. Intrauterine recordings taken in humans and animals have shown that the sounds of the mother’s vocalizations, breathing, heartbeat, body movements, footfalls, and digestion are all audible to the fetus (Parncutt, 2006). In-utero research and analysis has consistently shown that the fetus responds to the sound of the mother’s heartbeat. Sound measurements taken in the womb have shown that the sound of the mother’s heartbeat in the womb is 25db above the baseline noise (Querleu et al., 1988).

The sound and sensation of maternal footfalls as experienced by the fetus may provide information that informs the origin of the deep connection between dance and music. The interconnections between motor control and limbic structures mentioned above may provide the framework for retention of the tactile sensations of walking as felt in the womb in combination with the pulsing sounds of footfalls. The sonic-tactile association in the fetal brain will tend to be strong in keeping with the famous principle of neurological development that “neurons that fire together wire together”. Since adults take about 10,000 steps per day (Tudor-Locke and Myers, 2001) the presentation of the sound/sensory stimuli to the fetus is ubiquitous under normal circumstances. Music is often played at tempos similar to walking (Changizi, 2011) and it is reasonable to propose that one of the bases for this connection results from the concurrent stimuli of the tactile and sonic sensations of maternal footfalls informing the developing brain of the fetus.

The regular and constantly repeated sounds of maternal respiration create a repetitive acoustic framework that is broader and less specific than the heartbeat and possibly provides a basis for the recognition of more generalized pulse in music. Instruments that create amplitude contours that resemble that of a heartbeat such as drums made from stretched material over a resonating cylinder struck with a beater commonly keep the musical pulse.

The narrow spectrum of types of sounds that we humans respond to becomes apparent when we compare our time scale to other species. The ruby throated hummingbird has a resting heart rate of 615 beats per minute (Odum, 1945). If hummingbirds had their own music, it seems likely that it would be incomprehensively fast to our ears. The repetition rates of the pulses found in human music on the other hand (40–240 beats per minute) coincide with the slowest (respiration) and fastest (footfalls of running) pulses that can be heard in the womb. Human music seems to be built to the human scale.

The fetal environment provides little information that is significantly varied for the senses of sight, smell, or taste. Hearing is the exception to this dearth of information; *sounds* are always present. The patterns of speech heard in the womb are constantly changing and are available to be learned by the fetus. The heartbeat, although more consistent than speech or footfalls, also provides varied information that is constantly available to the fetus.

## The Drum

The drum is an example of an instrument that was invented and adopted for use in music in every culture. The similar construction of the drums from widely separated cultures demonstrate the common recognition of the sound of pulse that informed the development of the features of the instrument. The amplitude contour of the drum is such that it conforms to the sound of the pulse as heard from the womb. Over the centuries of the development of instruments, musicians found that stretching a skin over something round makes the sound die away more slowly (longer decay). It was also discovered that elongating the round skin holder into a cylinder made the sound more resonant and could give it a pitch (the tube resonates and lengthens the decay even more). And if it were struck with a cushioned beater the beginning of the sound would be less sudden (longer onset). Each of these modifications was made in order to create a pulse sound that we perceive as “good”. Recognition is the key point here. Why does a drum sound “better” than a stick hitting a table? The answer may be: because we *recognize* the drum sound, each of us who can hear heard it constantly for four months before we were born.

The construction of a drum enables it to create a heartbeat-like amplitude contour of the pulse instrument. Drums have been similarly constructed in many different cultures. (1) The onset of sound is graduated by a cushioned beater, a stretched animal skin, or both. (2) The decay of sound is elongated with a resonating chamber.

Geographically separated peoples came up with the same basic drum design. Three thousand years ago as the Greeks were drawing images on vessels of people playing the tympanon, the Chinese of the Shang Dynasty were making drums out of clay and stretched alligator skin. These parallel developments occurred well before the silk route had established a connection between the cultures of the East and West. Meanwhile the cross-rhythms of the Niger-Congo peoples were being played on Djembe drums that would not be seen by Europeans until the 15th century. That was around the time Westerners began invading the Americas where they found that drums had been made by all of the aboriginal Americans: from the Inuit and Ojibwe in the North, to the Lakota in the plains, the Aztecs between the continents and the Incas in the South.

To explain the parallel development in widely separated cultures of a musical instrument with a singular, basic design, and that was meant to be struck in repeating patterns of pulse and meter suggests a common root among all people. This commonality must be fundamental enough to supersede all linguistic, racial, and cultural differences. Furthermore, since all emotional responses begin with recognition, and since the preference for the sound of the drum is universal, then it is most logical to conclude that the template of sound that is recognized was formed in an environment that we all share. Emotional responses follow salient recognition; it would seem that the sound created by a drum triggers such recognition.

**FIGURE 1:**
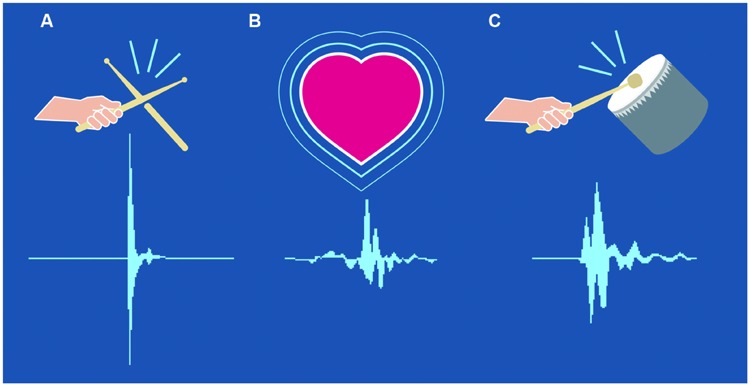
**Soundwaves created by (A) one stick hitting another, (B) a human heartbeat, (C) a drum struck with a cushioned beater**.

Below are a few figures and descriptions that outline the similarities between the amplitude contour of the human heartbeat and the drum:

**Figure 1A** above shows the spike of a transient sound made by one stick hitting another; this sounds “not so good” to us. For comparison, **Figure 1B** shows the amplitude contour of a human heartbeat. The amplitude contour of **Figure 1C** is created by a cushioned beater striking a drum. When skin is stretched over a round collar and placed over a resonating cylinder, the resonance of the cylinder amplifies and elongates the decay of the sound. Striking the skin with a cushioned beater creates a graduated onset. The resulting amplitude contour resembles that of the heartbeat as heard in the womb (onset 0.02 s, decay 0.06 s). In its completed form, when the onset is graduated as well as the decay, this sounds “just right” to us. The feelings of “good”, or “just right”, like all emotional responses, begin with recognition. It is possible that we recognize the sound that was imbued in our growing brains.

Tactile response is present in contemporary music that is presented in high decibel levels. The musicians of these genres are seeking sound levels that are not only able to be heard, but are also able to be felt. In an interview with the author, Dickie Peterson, one of the original band members of a predecessor of heavy metal music, reported that they wanted to create music that could be felt. The desire for tactile sense of music may be traced to the sensations of the womb. The maternal heartbeat creates a pressure wave that not only can be clearly heard but may also be felt by the fetus. The dense, liquid-filled environment of the womb allows for a pressure wave emanating from the maternal heartbeat to travel through the tissue of her womb into and through the body of the fetus.

## Meter

The differently paced and constantly overlapping pulses of heartbeats, inhalation, and exhalation create patterns of strong and weak beats. In the womb the inhalation is louder than the exhalation. When the inhalation sounds simultaneously with the heartbeat, the combination forms the strongest sound, exhalation with the heartbeat is somewhat strong, and the heartbeat alone is weakest. As a result, it is reasonable to conclude that music has evolved to include repeating patterns of strong and weak beats that resemble those in the fetal environment. The combination of heartbeats and breathing heard in the womb may be the basis for musical meters.

From this perspective, the combinations of strong and weak pulses found in the primary meters would be derived from the sounds of respiration combined with the sound of the heartbeat. Strong-weak is duple meter. **ONE** two THREE four (1 strongest - 2 weak - 3 strong - 4 weak) is known as “common time” in Western music. When respiration and heartbeat are combined: 1 inhalation + heartbeat – 2 heartbeat alone - 3 exhalation + heartbeat - 4 heartbeat alone, the result is common time and this is consistent with normal human heart and respiratory rates (approximately four heartbeats/respiratory cycle).

The prevailing duality of pulse in Western music is the same duality found in the human rhythms of heartbeats, breathing, and walking. When respiration and heart beat are combined we have (Table 2):

**Table 2:** Strong and weak beats resulting from combined respiration and heartbeats.

**ONE**	inhalation + heartbeat
Two	heartbeat alone
THREE	exhalation + heartbeat
Four	heartbeat alone

Or another way to see it:


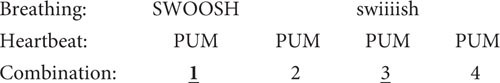


Perhaps common time is common to us all. Theoretically, the possible combinations of tempos and stresses that are available are infinite, yet the spacing, stresses, and tempos of common time are the very same as the combined pulsations that are often heard in the womb. Other meters that are used in music can also be traced to other combinations of the strong and weak beats created intermittently by the varied combinations of heartbeat and respiration. The ratio of (1) STRONG – (2) weak is 2/4 time, and (1) STRONG – (2) weak – (3) weak is 3/4 time.

Footfalls also create pulses that can be heard and felt by the fetus and may combine with the heartbeat to create overlaps that also augment the strength of some beats. Naturally, not all mothers have the same heart/respiration ratios and the synchronicity of the beats is constantly changing. The heartbeat is faster when walking and the pressure waves created by the footsteps of a pregnant mother are audible to the fetus (Parncutt, 2006). The faster pace of the heartbeat combined with the even pulses of footfalls creates a relatively quick 2/4 m. Even the rarely heard footfalls of running may be found in the developing brain’s list of recognized combined pulses.

A weak beat placed where the silence occurs between the duple pulses of the heart creates a triple meter. Here is a visual approximation using “lubb, dub”, the traditional vocal approximation of the sound of the heartbeats used by physicians:


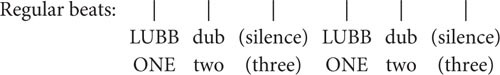


These mixed meters may also traceable to the fetal sonic environment. 6/8 is a combination of triple/duple (**1** 2 3 **4** 5 6) combining the LUBB – dub - silence of a quickened heartbeat with inhalation and exhalation.

It has been noted that the music of some cultures such as some Balkan cultures have music that does not use common meters. Irregular meters may also be derived from the combination of heartbeat and respiration. It should be noted that symmetrical rhythms are not common, even in Balkan music; the rhythms of Balkan music are primarily duple. Generally there is a distinction between underlying pulse rhythm and melodic rhythm, but this may be a case where they mingle. The Balkan exception may be due to the melodic rhythm in the language, since Greek allows for more irregularities between stressed and unstressed syllables than English (Patel, 2008).

## Melody

The maternal voice heard by the fetus in the womb may provide the foundation of musical notes and melody. The pitches created by the vocal chords of the mother are distinct and loud in the fetal environment. A team of researchers from the University of Florida headed by Douglas Richards managed to convince eight bedridden mothers-to-be to have microphones inserted into the uterus and placed near the head of the fetus. The mothers were asked to speak in a loud voice as the intrauterine sound level was recorded. They found that the average mother’s voice in the womb is 77.2 dB, nearly four times greater than the intensity measured in the air at a distance of 24 inches (Richards et al., 1992). A spoken sentence is heard in the womb as a pattern of discrete pitches in a variety of melodic contours and rhythms.

## Discrete, Single-Frequency Segments (Musical Notes)

Discrete, single-frequency segments (notes) are found in the music of all cultures. The mother’s speech that is heard in the womb consists primarily of single-frequency segments created by the vowels between the consonants (Querleu et al., 1988). These units may provide the singular basis for notes in music. We do not find music from any culture that consists primarily of sliding pitches. Mammalian vocalizations generally consist of syllables that have contoured frequencies (sliding pitches) such as a cat’s meow or a dog’s submissive whimper as well as the human vocalizations such as moaning and weeping (Parvizi et al., 2001). Despite this preference for contoured frequencies in emotional vocalizations, human music contains a preponderance of discrete single-frequency units.

This variance between the characteristics of emotionally generated vocalizations and the characteristics of music might be explained by the womb origin of music. The prevalence of discrete single-frequency segments is a feature of the music of all cultures. One of the prominent features of Schubert’s melodic style is that he gave each syllable in the lyrics only one note. Irving Berlin, who was described by George Gershwin as “America’s Schubert” used the same one-to-one note/melody standard as Schubert. Accordingly, most languages are made up of predominantly single-pitch segments separated by consonants.

The preference for discrete single-frequency segments in music may be accounted for when acoustical properties of the womb are considered. The middle and lower frequencies of the mother’s speech emanating from the maternal vocal chords are carried directly to the ears of the fetus through the medium of the liquid and tissue of the womb that is approximately five times more efficient than air. The high frequency sounds of the consonants such as “ch”, “t”, “s”, and “sh” formed at the opening of the mouth are significantly attenuated when transferred from the air to the tissue and further attenuated by the absorption of sound by the surrounding tissues in the womb. Due to this attenuation, the consonants of speech are nearly inaudible in the womb but the “melody” of the pitches created by the vowels between the consonants is clearly audible and would sound something like humming discrete single-frequency pitches.

When we compare the features of maternal speech as it is heard in the womb to the features of melodies that are found in the music of a wide variety of cultures we find compelling similarities. Indeed, it is a supportable assertion that the contours and prosody of specific languages as heard by the fetus are nearly identical to the melodic contours of the music associated with that language. All of the commonly found features of musical melodies are present in the mother’s voice as it is heard in the womb. Speech is produced in predominantly consonant intervals and contains implied tonalities (Schwartz and Purves, 2004; Bowling et al., 2009). As a consequence, the melodies heard in the womb are primarily harmonically consonant.

The prosody of languages may form the bases for melodic treatment in music. Newborns of French mothers prefer the sound of the French language to Russian (Mehler et al., 1988). The newborns still prefer the French language when the speech is filtered to remove the consonant and vowel sounds in order to present maternal speech as it is heard from inside the mother, retaining only the melody, but they do not show a preference for the melody of the French language when played backward, implying that a fetus is able to recognize intervallic relationships and melodic contours.

Evidence for fetal learning of the melodic contours of maternal speech is also found in the cries of newborns that emulate the melodic contours of the mother’s language (Mampe et al., 2009). Kathleen Wermke of the University of Würzburg in Germany studied five-day-old infants and discovered that they use the contours of their own mothers’ language in their cries (Mampe et al., 2009). Words and combinations of words create recognizable rhythms that are found in the melodic rhythms of musical motives. Cultures whose languages have accented syllables also have corollary accents in their melodies. For example, the definite articles in the Germanic and Romance languages (*the* sea, *die* See, *la* mer) are heard in the musical upbeats at the beginning of many melodies. The music of cultures whose languages do not contain definite articles, rarely have musical upbeats to their melodies. Note the preference for beginning melodies on the beat in the music of Mussorgsky (Russian) and Dvorak (Czech). A number of other commonalities have been found between the melodic rhythms of a culture and the speech rhythms in its language (Huron and Ollen, 2003; Patel and Daniele, 2003).

## Frequency Range of Melodic Instruments

The womb origin of melody might also explain the range of the most commonly used melodic instruments. The frequency range of melodic instruments in a wide variety of cultures is roughly 200–900 Hz, the same as the frequency range of an adult human female voice. The following instruments have strings tuned to pitches in this range: the West African kora, the Chinese guqin, the Indian sitar, the North American Apaches’ tsii’edo’a’tl, and the European violin. The flute may be even more ubiquitous. Fossilized bone flutes have been found that date back more than 30,000 years (Atema, 2004). Many musical instruments have been invented and modified over the years to create the kinds of resonance-enhanced richness that remind us of the human voice.

## Continuity

The underlying beats may also provide another hidden universal characteristic of music: nearly all of the music heard in all cultures is continuous. While speech stops and starts, music rarely presents any other than an unbroken stream of sound. Continuity is key to musical appreciation, attention, and subconscious recognition. The acoustic environment of the womb also provides a constant stream of sound that could provide the foundation of recognition that music builds upon. Even when melodies are presented in phrases that are separated from one another, there is continuity in the underlying pulse and accompanimental patterns. In the fetal environment, the maternal voice comes and goes, but the pulse and meter always remain. There are exceptions to the continuity of music, but the silence is typically brief and the tempo of the pulse usually remains.

## Tests of the Prenatal Roots of Music

To test whether the elements of music are based upon the sounds of the environment during brain development, I conceptualized two studies that compared the responses of a species to music composed for humans and music composed specifically for that species. These studies were carried out by Dr. Charles Snowdon and colleagues at the University of Wisconsin-Madison. If the concept is correct, it should be possible to create music that would be effective for another species that is based upon the sounds that are present as the brain structures of the limbic system of that species are being formed.

The first study compared the responses of cotton-top tamarin monkeys to four types of music: aggressive human music, calming human music, aggressive tamarin music, and calming tamarin music (Snowdon and Teie, 2010, 2013). In addition to the use of instrumental music that was based on vocalizations of each species, I included in the calming music for the tamarin monkeys a pulse that was equal in pacing to the resting heart rate of an adult monkey (200-220 beats per minute). Since the brain of a monkey is 60% of its adult size at birth I included instrumental representations sounds of the womb in the music for the tamarins. As had been the case in most other tests of the effects of human music on other species, the tamarins were indifferent to the human music (McDermott and Hauser, 2007). The calming tamarin music was effective in calming the monkeys and the arousing music led to increased movement and behavior indicative of anxiety. There was one significant exception to the tamarin’s indifference to the human music: the only response that was elicited by the human control music was that they were calmed by the aggressive human heavy metal music that has a pulse of 200 beats per minute. This apparent anomaly supports the proposition that the sonic characteristics of the maternal pulse may have a calming effect when presented in music.

The second study was conducted on cats (Snowdon et al., 2015). Since the brain of a newborn cat is only 1/8 the size of what it will be at 10 weeks, the sounds of the womb were presumed to be not salient for cats. A reward-related sound that is present during the development of the cat’s brain is the sound of suckling. The music for cats, therefore, included musical instruments designed to resemble the sound of suckling as it would be heard by a nursing kitten. The data on the effect of species-specific music on cats from this study were even stronger than in the tamarin monkey study.

## Probability that the Features Common to the Womb and Music are Coincidental

A demonstration of the statistical probability that these parameters are connected in a causal relationship would strongly support the theory of the prenatal roots of music. In order to assess that probability it is necessary to deduce the probability that the similarities between the elements of music and the sounds present in the fetal environment are due to coincidence. The first consideration to be accounted for is the number of variables. How many variable features are there in each element? Below is a brief outline of the variables for each element.

Pulse:

• The pulse could be irregular with any number of different variations in the durations between them.
• The pulse could be from minutes to milliseconds apart.
• There could be no pulse/beat at all; music could have regular or irregular squeaks, or gurgles, or swishes, etc.

Meter:

• The combinations of the accented beats that comprise meters create an infinite number of possible patterns.

Frequency range of melodic instruments:

• The frequency range of melodic instruments would most likely center on our most sensitive hearing range that is used to produce consonants: from 2-4 kHz. Despite this sensitivity, our melodies are usually in the fairly narrow range of 200 to 800 Hz. (This is noted to refute the common assumption that the most common melodic instruments sound in the frequency range of the treble register because it is easier for people to hear in that range).

The determination of coincidence would also need to include the probability that the combination of the above-listed variables is coincidental. Finally, it would be necessary to assess the probability that these elements are similar in not only the music of one culture, but in the music of every culture.

Since it is impossible to provide data on the probabilities that a given sonic element would or would not be present in either music or the fetal environment, it is also impossible to present the possibility of the coincidence of the elements as a statistical probability. However, given the large number of variables and the ubiquity of the commonalities between the sounds of the womb and the music of all cultures, the probability that they are similar by coincidence would seem to be astronomically remote.

## Conclusion

The questions of the origins of music presented at the beginning of this article are plausibly answered by the prenatal environment. There appears to be a correlation between the acoustic features of the common elements of music found in diverse and widely separated cultures and the acoustic features of sounds that are present in the womb. The neurological development of the fetus allows for the absorption and retention of those sounds to be implanted as templates of recognition in the brain.

I propose that the acoustic parameters of the sounds in the womb are the same as the parameters of universal characteristics of music. It is possible to match, one-for-one, the sounds of the womb to those elements that can be found in the music of all cultures.

## Author Contributions

The author confirms being the sole contributor of this work and approved it for publication.

## Conflict of Interest Statement

The author declares that the research was conducted in the absence of any commercial or financial relationships that could be construed as a potential conflict of interest.
